# Sarcopenia Exacerbates Obesity-Associated Insulin Resistance and Dysglycemia: Findings from the National Health and Nutrition Examination Survey III

**DOI:** 10.1371/journal.pone.0010805

**Published:** 2010-05-26

**Authors:** Preethi Srikanthan, Andrea L. Hevener, Arun S. Karlamangla

**Affiliations:** 1 Division of Gerontology, Department of Medicine, David Geffen School of Medicine, University of California Los Angeles, Los Angeles, California, United States of America; 2 Division of Endocrinology, Diabetes, and Hypertension, Department of Medicine, David Geffen School of Medicine, University of California Los Angeles, Los Angeles, California, United States of America; Pennington Biomedical Research Center, United States of America

## Abstract

**Background:**

Sarcopenia often co-exists with obesity, and may have additive effects on insulin resistance. Sarcopenic obese individuals could be at increased risk for type 2 diabetes. We performed a study to determine whether sarcopenia is associated with impairment in insulin sensitivity and glucose homeostasis in obese and non-obese individuals.

**Methodology:**

We performed a cross-sectional analysis of National Health and Nutrition Examination Survey III data utilizing subjects of 20 years or older, non-pregnant (N = 14,528). Sarcopenia was identified from bioelectrical impedance measurement of muscle mass. Obesity was identified from body mass index. Outcomes were homeostasis model assessment of insulin resistance (HOMA IR), glycosylated hemoglobin level (HbA1C), and prevalence of pre-diabetes (6.0≤ HbA1C<6.5 and not on medication) and type 2 diabetes. Covariates in multiple regression were age, educational level, ethnicity and sex.

**Principal Findings:**

Sarcopenia was associated with insulin resistance in non-obese (HOMA IR ratio 1.39, 95% confidence interval (CI) 1.26 to 1.52) and obese individuals (HOMA-IR ratio 1.16, 95% CI 1.12 to 1.18). Sarcopenia was associated with dysglycemia in obese individuals (HbA1C ratio 1.021, 95% CI 1.011 to 1.043) but not in non-obese individuals. Associations were stronger in those under 60 years of age. We acknowledge that the cross-sectional study design limits our ability to draw causal inferences.

**Conclusions:**

Sarcopenia, independent of obesity, is associated with adverse glucose metabolism, and the association is strongest in individuals under 60 years of age, which suggests that low muscle mass may be an early predictor of diabetes susceptibility. Given the increasing prevalence of obesity, further research is urgently needed to develop interventions to prevent sarcopenic obesity and its metabolic consequences.

## Introduction

Obesity and type 2 diabetes constitute a significant health care concern in the United States and other developing and developed nations, especially since their incidence is on the rise in children and young adults. Sarcopenic obesity, the co-existence of sarcopenia and obesity, [1] is seen in 5–10% of healthy, ambulatory, community-dwelling Americans in their sixties, rising to over 50% in those over age eighty [2]. Studies indicate that up to 50% of muscle may be lost by the age of 90 years[3]. Since muscle is the primary tissue contributing to whole-body insulin-mediated glucose disposal, sarcopenia may be an important causal factor in age-induced insulin resistance and type 2 diabetes susceptibility.

Inflammation is a central underpinning in the pathogenesis of insulin resistance and is also seen in both obesity and sarcopenia. Inflammation may be an important mediator in restraining myogenesis and/or accelerating muscle protein degradation. In addition, intramyocellular lipid accumulation, seen in obesity, results in the formation of bioactive lipid intermediates and lipid peroxides, which are known to activate pro-inflammatory cascades [4]. Furthermore, myokines secreted by skeletal muscle [5] have been found to prevent inflammation and insulin resistance, thus counteracting the pro-inflammatory and metabolic effects of adipokines produced in adipose tissue; the relative paucity of myokines relative to adipokines in sarcopenic obesity may increase the risk of metabolic and cardiovascular disease[6].

Recent studies in rodents suggest a strong inverse association between muscle mass and disease risk. Even a modest increase in muscle mass can prevent diet-induced obesity and insulin resistance as well as atherogenesis in prone mice[5],[7]. Consistent with this, Stephen et al. found a positive association between sarcopenic obesity and cardiovascular disease in the older adults from the Cardiovascular Health Study [8]. However, since type II muscle fibers, described as glycolytic and insulin resistant [9], are lost to a greater extent than type I fibers [10] in age-related muscle atrophy, sarcopenia could theoretically also increase insulin sensitivity and cause some beneficial alterations in glucose metabolism in older adults [11].

Accordingly, we hypothesize that sarcopenic obese individuals have more insulin resistance and higher prevalence of dysglycemia (i.e., impaired glucose tolerance and diabetes), than individuals with neither sarcopenia nor obesity, those with obesity alone (without sarcopenia), and those with sarcopenia alone (without obesity). We further hypothesize that this association will be stronger in young and middle aged adults than in older adults, in whom sarcopenia may mean a preferential reduction in insulin-resistant fibers.

To test this hypothesis, we assessed the level of insulin resistance and dysglycemia in sarcopenic obese individuals, obese individuals without sarcopenia, sarcopenic individuals without obesity, and in those with neither sarcopenia nor obesity, in a nationally representative sample, and tested for effect modification by age.

## Methods

### Ethics statement

Written informed consent was obtained from all participants, and the protocol was approved by the institutional review board of the National Center for Health Statistics, and the study procedures were carried out in accordance with the principles of the Declaration of Helsinki.

### Design and Methods

The National Health and Nutrition Examination Survey (NHANES) III was a national survey conducted from 1988 through 1994, using a stratified, multistage, probability cluster design. The total sample included 33,199 persons[12] of whom, 17,756 were older than 20 years and non-pregnant. The full evaluation included a standardized home interview (with a medication review), a physical examination in a mobile examination center, and a fasting blood draw.

Our analytic sample (N = 14,528) was restricted to those who were 20 years or older and non-pregnant, and had measurements of bioelectrical impedance (BI), height, and body weight, the three variables we utilized to indirectly estimate the presence of sarcopenic obesity. Participants who had cardiac pacemakers or had previously undergone limb amputation were excluded from the measurement of BI [13].The analytic sample included 2370 women and 2284 men who were 60 years of age or older.

### Measurements: Exposures

Body height and weight were measured, and converted to body mass index (BMI) in units of kg/meter-squared. Obesity was defined as BMI >30 kg/m^2^. Waist size and hip size were measured by standard protocols and waist-to-hip circumference ratio was created. BI was measured using the Valhalla Scientific Body Composition Analyzer 1990 B [14], and used to estimate skeletal muscle mass (in kg) via the BI analysis equation of Janssen et al [15].
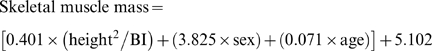
with height measured in cm, BI measured in ohms, sex coded 1 for men and 0 for women, and age measured in years. Muscle mass (in kg) was divided by body mass (in kg) and multiplied by 100% to create skeletal muscle index (SMI). Similar to the approach used to identify osteoporosis from bone mineral density [16], sarcopenia is defined as SMI more than two standard deviations below the sex-specific, young adult (ages, 18-39) means: 31.0% in men and 22.0% in women [17].

### Measurements: Outcomes

Serum insulin and plasma glucose were measured from fasting blood samples (if fasted 6 hours or more) using radioimmunoassay and a hexokinase enzymatic method, respectively [13], and used to calculate insulin resistance by the Homeostasis model assessment of insulin resistance (HOMA-IR) which is approximated using the formula below:
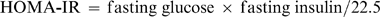
with glucose in mmol/L and insulin in μU/ml, for participants whose fasting plasma glucose ranged from 3.0 to 25.0 mmol/l and fasting insulin ranged from 3 to 55 µU/ml[18]. HOMA-IR data were available for 12,046 subjects. Glycosylated hemoglobin (HbA1c) was measured using an ion-exchange high-performance liquid chromatography method using the Diamat Analyzer System, and used to define *dysglycemia* based on standard HbA1C thresholds [19]. Specifically, *diabetes (DM)* was defined as one or more of 1) HbA1C ≥6.5% 2) Fasting glucose ≥7 mmol/L (126 mg/dl), 3) self report of DM and /or 4) use of DM medication (oral hypoglycemic agents and /or insulin), and *pre-diabetes* was defined by 1) HbA1C ≥6% but <6.5% **OR **fasting ≥5.5 mmol/L(100 mg/dL) but <7.0 mmol/L(126 mg/dL), 2) no self-reported DM, **and** 3) absence of DM medications.

### Measurements: Covariates

Age, race/ethnicity (non-Hispanic white, non-Hispanic black, Mexican American, and other), and completed years of education and sex were obtained from self reports. Serum C-reactive protein (CRP) concentration was measured using latex-enhanced nephelometry with a Behring Nephelometer Analyzer System (Behring Diagnostics Inc)[20]. Details about the laboratory procedures and quality control have been published [20]. Serum CRP levels greater than 10 mg/dL were set to missing to avoid capturing acute elevations in CRP due to infectious causes.

### Statistical Analyses

To study the association between sarcopenic obesity and insulin resistance/dysglycemia, we examined four outcomes (HOMA-IR, HbA1C, prevalence of pre-diabetes, prevalence of diabetes mellitus) in sarcopenic obese individuals, sarcopenic non-obese individuals, obese non-sarcopenic individuals, and those with neither sarcopenia nor obesity (the reference group). To control for confounders (namely age, sex, education, and race/ethnicity), we used multivariable logistic regression for the two prevalence outcomes (diabetes and pre-diabetes) and multivariable linear regression for the continuous outcomes: HbA1C and HOMA-IR; these variables were log-transformed before model fitting. To minimize residual confounding by age, we included age both as a continuous and a categorical variable (20–29 y, 30–39 y, 40–49 y, 50–59 y, 60–74 y, and ≥75 y). We similarly, included years of education both as a continuous and a categorical variable (<12 y, 12–14 y, 15–17 y, and >17 y, intended to capture the effect of credentialing at high school and college). We also repeated the analyses after excluding diabetics to minimize confounding by reverse causation (i.e., diabetes leading to sarcopenia and/or obesity).

We tested for effect modification by age (dichotomized: <60 years vs. > = 60 years) and gender by including interaction terms in the models for each of the four dependent variables. Based on the results of the interaction testing, we conducted further stratified analyses. In supplementary analyses, to test if sarcopenia/obesity associations with inflammation are consistent with their dysglycemia associations, we also examined serum CRP level (after log-transformation) as a continuous outcome in multivariable linear regression.

We used SAS, release 9.2 (SAS Institute Inc, Cary, NC) for all the analyses.

## Results

The study sample was representative of the complete NHANES sample that was non pregnant and 20 years or older (Table 1), except that the study participants were younger, less frequently male, non-Hispanic White, and diabetic than those excluded from the study. Those excluded had similar BMI, HOMA-IR, and HbA1C as those in the study sample. The average age of participants in the study sample was 45 years, 51.7% were female and 42.2% were non-Hispanic Whites.

**Table 1:** Descriptive statistics (median with inter-quartile range, or percentage).

	*Study Sample*			
	Complete sample(N = 14,528)	Under 60 years(N = 9,892)	60 years or older(N = 4,636)	*Excluded* *(N = 3,228)
Age (years)	45.0(32.0 to 64.0)	37.0 (29.0 to 45.0)	71.0 (65.0 to 78.0)	66.0 (41.0 to 80.0)
Body mass index (kg/m^2^)	26.3(23.2 to 30.0)	26.2 (23.0 to 30.2)	26.5 (23.6 to 29.8)	26.3 (22.6 to 30.4)
Skeletal muscle index (%)	33.9 (27.9 to 39.4)	35.0 (29.2 to 40.5)	31.0 (25.8 to 36.9)	-
Glycosylated hemoglobin (%)	5.40 (5.00 to 5.7)(n = 14026)	5.20 (4.90 to 5.60)(n = 9537)	5.60 (5.30 to 6.10)(n = 4489)	5.50 (5.10 to 5.90)(n = 1622)
HOMA-IR (mg/dl × μU/ml)	2.12 (1.45 to 3.30)(n = 12046)	2.00 (1.38 to 3.10)(n = 8173 )	2.38 (1.61 to 3.67)(n = 3873)	2.25 (1.46 to 3.46)(n = 1031)
Gender: Male	48.3%	47.9%	49.2%	50.3%
NH White	42.2%	34.6%	58.2%	51.2%
NH Black	27.3%	31.0%	19.6%	24.9%
Hispanic	26.4%	29.8%	19.0%	21.8%
Other	4.18%	4.60%	3.28%	2.23%
Sarcopenic without obesity	1.14%	0.18%	3.17%	-
Obese without sarcopenia	21.0%	22.9%	17.1%	-
Sarcopenic obesity	4.50%	3.39%	6.9%	-
Pre-diabetes	25.6%	20.6%	35.8%	22.9%
Diabetes	13.9%	8.7%	24.4%	35.0%

*Those in the NHANES III sample who were older than 20 years and not pregnant, but were excluded because they were missing bioelectrical impedance or body mass index measurement.

Sarcopenia was more prevalent in obese than non-obese participants (4.5% vs. 1.14%), and this was true both in those under 60 years of age (3.4% vs. 0.2%) and in those 60 years and older (6.9% vs. 3.2%). Comparing participants under 60 years of age with those 60 years or older in the study sample, the older group had lower SMI and more sarcopenia without obesity but less obesity without sarcopenia. Yet, the older adults had more insulin resistance and dysglycemia than the younger group: Median HbA1C, HOMA IR, and prevalence of pre-diabetes and DM were all higher in older adults.

In the complete sample, adjusted for age, sex, educational level (both as a continuous and categorical variable) and race/ethnicity, sarcopenia (without obesity) was significantly associated with increased HOMA-IR and pre-diabetes, but not with dysglycemia or diabetes outcomes, and in particular there was a marginally significant association with *decreased* risk of DM (Table 2). In contrast, obesity with and without sarcopenia was associated positively with all four outcomes. However, consistent with our main hypothesis, participants with sarcopenic obesity had significantly higher index of insulin resistance (HOMA-IR ratio 1.16, 95% CI 1.12 to 1.18, p<0.001) and chronic hyperglycemia (HbA1C ratio 1.021, 95%% CI 1.011 to 1.043, p = 0.002) than obese, non-sarcopenic participants, but they did not have higher prevalence of pre-diabetes and DM. This pattern of associations was essentially unchanged when diabetics were excluded to reduce confounding by reverse causation (i.e. diabetes leading to sarcopenia or obesity) - See Table 2.

**Table 2:** Associations of insulin resistance and dysglycemia with sarcopenia, obesity, and sarcopenic obesity, adjusted for age, sex, race, and education.

Outcomes:Effect size:	Insulin resistance HOMA-IR ratio^1^ (95% CI)p value	Glycosylated hemoglobin HbA1C ratio^2^ (95% CI)p value	Pre-diabetes Odds Ratio^3^ (95% CI)p value	Diabetes Odds Ratio (95% CI)p value
Sarcopenia without obesity	1.39 (1.26 to 1.52)p<.0001	1.00 (0.97 to 1.02)p = 0.7	1.43 (1.02 to 2.01)p = 0.04	0.78 (0.50 to 1.23)p = 0.3
Obesity without sarcopenia	1.84 (1.80 to 1.89)p<.0001	1.054 (1.048 to 1.061)p<.0001	1.44 (1.30 to 1.59)p<.0001	2.44 (2.16 to 2.76)p<.0001
Sarcopenic Obesity	2.13 (2.02 to 2.23)p<.0001	1.075 (1.061 to 1.088)p<.0001	1.46 (1.21 to 1.75)p<.0001	2.81 (2.30 to 3.43)p<.0001
Sarcopenia without obesity - diabetics excluded	1.37 (1.26 to 1.50)p<.0001	1.00 (0.99 to 1.02)p = 0.6		
Obesity without sarcopenia - diabetics excluded	1.75 (1.71 to 1.79)p<.0001	1.025 (1.021 to 1.029)p<.0001		
Sarcopenic Obesity - diabetics excluded	1.99 (1.89 to 2.08)p<.0001	1.035 (1.027 to 1.044)p<.0001		

1 Ratio of HOMA IR in sarcopenic obese group to HOMA IR in reference group (neither sarcopenic nor obese) where HOMA IR is the Homeostatic Model Assessment of Insulin Resistance.

2 Ratio of HbA1C in sarcopenic obese group to HbA1C in reference group (neither sarcopenic nor obese) where HbA1C is the blood level of glycosylated hemoglobin.

3 Pre-diabetes is defined as a 1) HbA1C ≥6% but <6.5%, OR fasting glucose ≥5.5 but <7 mmol/L, 2)no self-reported DM, **and** 3) absence of DM medications.

In interaction testing, age (dichotomized at 60 years) modified effects of sarcopenia and obesity (Table 3). All interactions between gender and sarcopenia/obesity were non significant at the 0.1 level (data not shown).

**Table 3:** P values for interactions of age (<60 years vs. ≥60 years) with sarcopenia, obesity, and sarcopenic obesity in models adjusted for age, sex, race, and education.

Outcomes:Effect size:	Insulin resistanceHOMA-IR ratio^1^	Glycosylated hemoglobin HbA1C ratio^2^	Pre-diabetes Odds Ratio^3^	Diabetes Odds Ratio
Sarcopenia without obesity	0.13	0.01	0.4	0.07
Obesity without sarcopenia	<.0001	0.3	0.003	0.04
Sarcopenic Obesity	<.0001	<.0001	0.04	0.0005
Sarcopenia without obesity - diabetics excluded	0.2	0.3		
Obesity without sarcopenia - diabetics excluded	<.0001	0.6		
Sarcopenic Obesity diabetics excluded	<.0001	<.0001		

1 Ratio of HOMA IR in sarcopenic obese group to HOMA IR in reference group (neither sarcopenic nor obese) where HOMA IR is the Homeostatic Model Assessment of Insulin Resistance.

2 Ratio of HbA1C in sarcopenic obese group to HbA1C in reference group (neither sarcopenic nor obese) where HbA1C is the blood level of glycosylated hemoglobin.

3 Pre-diabetes is defined as a 1) HbA1C ≥6 but <6.5%, OR fasting glucose ≥5.5 but <7 mmol/L, 2)no self-reported DM, **and** 3) absence of DM medications.

In age-stratified analyses, associations with insulin resistance and dysglycemia were stronger in the younger group (See Tables 4 and 5). In those under 60 years of age, sarcopenia without obesity was associated with higher HOMA-IR and HbA1C, but not with pre-diabetes and DM prevalence (see Table 4). However, the odds ratio confidence intervals for the latter two outcomes were unusually wide, which may reflect reduced power due to the small number of sarcopenic non-obese participants in the younger age group. In comparison to obese, non-sarcopenic participants, younger participants with sarcopenic obesity had significantly higher HOMA-IR (HOMA-IR (ratio 1.26, 95% CI 1.22 to 1.31, p = <.0001), higher HbA1C (ratio 1.054, 95% CI 1.032 to 1.062, p <.0001), and higher prevalence of DM (odds ratio 1.54, 95% CI 1.44 to 1.65, p = <.0001).

**Table 4:** Associations of insulin resistance and dysglycemia with sarcopenia, obesity, and sarcopenic obesity, in adults younger than 60 years, adjusted for age, sex, race, and education.

Outcomes:Effect size:	Insulin resistance HOMAIR ratio^1^ (95% CI)p value	Glycosylated hemoglobin HbA1C ratio^2^ (95% CI)p value	Pre-diabetes Odds Ratio^3^ (95% CI)p value	Diabetes Odds Ratio (95% CI)p value
Sarcopenia without obesity	1.67 (1.27 to 2.20)p = 0.0003	1.086 (1.024 to 1.155) p = 0.02	0.77 (0.21 to 2.79) p = 0.7	2.39 (0.64 to 8.96)p = 0.2
Obesity without sarcopenia	1.90 (1.84 to 1.95)p<.0001	1.053 (1.045 to 1.060) p<.0001	1.62 (1.43 to 1.84)p<.0001	2.73 (2.30 to 3.24)p<.0001
Sarcopenic Obesity	2.39 (2.24 to 2.55)p<.0001	1.10 (1.09 to 1.12) p<.0001	1.81 (1.39 to 2.36)p<.0001	4.20 (3.31 to 5.34)p<.0001
Sarcopenia without obesity - diabetics excluded	1.58 (1.20 to 2.06)p = 0.001	1.033 (0.983 to 1.081) p = 0.2		
Obesity without sarcopenia - diabetics excluded	1.80 (1.75 to 1.85) p<.0001	1.026 (1.021 to 1.030) p<.0001		
Sarcopenic Obesity - diabetics excluded	2.20 (2.06 to 2.35)p<.0001	1.051(1.039 to 1.063) p<.0001		

1 Ratio of HOMA IR in sarcopenic obese group to HOMA IR in reference group (neither sarcopenic nor obese) where HOMA IR is the Homeostatic Model Assessment of Insulin Resistance.

2 Ratio of HbA1C in sarcopenic obese group to HbA1C in reference group (neither sarcopenic nor obese) where HbA1C is the blood level of glycosylated hemoglobin.

3 Pre-diabetes is defined as a 1) HbA1C ≥6% but <6.5%, OR fasting glucose ≥5.5 but <7 mmol/L, 2) no self-reported DM, **and** 3) absence of DM medications.

**Table 5:** Associations of insulin resistance and dysglycemia with sarcopenia, obesity, and sarcopenic obesity, in adults 60 years or older, adjusted for age, sex, race, and education.

Outcomes:Effect size:	Insulin resistance HOMAIR ratio^1^(95% CI)p value	Glycosylated hemoglobin HbA1C ratio^2^(95% CI)p value	Pre-diabetes Odds Ratio^3^ (95% CI)p value	Diabetes Odds Ratio (95% CI)p value
Sarcopenia without obesity	1.34 (1.20 to 1.49)p<.0001	0.99 (0.96 to 1.02) p = 0.3	1.50 (1.05 to 2.14)p = 0.03	0.71 (0.44 to 1.16)p = 0.2
Obesity without sarcopenia	1.70 (1.61 to 1.79)p<.0001	1.062 (1.041 to 1.073) p<.0001	1.12 (0.94 to 1.33) p = 0.2	2.09 (1.74 to 2.51) p<.0001
Sarcopenic Obesity	1.86 (1.73 to 2.00)p<.0001	1.042 (1.021 to 1.054) p<.0001	1.16 (0.90 to 1.49) p = 0.25	2.10 (1.62 to 2.73) p<.0001
Sarcopenia without obesity - diabetics excluded	1.33 (1.21 to 1.46)p<.0001	1.00 (0.99 to 1.02) p = 0.96		
Obesity without sarcopenia - diabetics excluded	1.60 (1.52 to 1.67)p<.0001	1.021(1.011 to 1.034) p<.0001		
Sarcopenic Obesity - diabetics excluded	1.74 (1.67 to 1.87)p<.0001	1.012(1.001 to 1.033)p = 0.001		

1 Ratio of HOMA IR in sarcopenic obese group to HOMA IR in reference group (neither sarcopenic nor obese) where HOMA IR is the Homeostatic Model Assessment of Insulin Resistance.

2 Ratio of HbA1C in sarcopenic obese group to HbA1C in reference group (neither sarcopenic nor obese) where HbA1C is the blood level of glycosylated hemoglobin.

3 Pre-diabetes is defined as a 1) HbA1C ≥6% but <6.5%, OR fasting glucose ≥5.5 but <7 mmol/L, 2) no self-reported DM, **and** 3) absence of DM medications.

In contrast, in those 60 years and older, sarcopenia without obesity was associated with higher HOMA-IR and higher prevalence of pre-diabetes but not with HbA1c or prevalence of DM (see Table 5). Moreover, although sarcopenic obesity (compared to the non-sarcopenic, non-obese referent) was significantly associated with HOMA-IR, HBA1C, and DM outcomes, older sarcopenic obese individuals did not significantly differ in any of the outcomes from older obese, non-sarcopenic individuals.

To determine whether sarcopenia/obesity associations with inflammation in the two age groups are in concordance with their associations with insulin resistance and dysglycemia, we examined log CRP as outcome in parallel linear regression models. In those under 60 years of age, sarcopenia was independently associated with increased CRP in both non-obese (CRP ratio 1.13, 95% CI 1.01 to 1.30, p = 0.02) and obese individuals (CRP ratio 1.093, 95% CI 1.041 to 1.143, p = 0.002). In those 60 years or older, sarcopenia was not independently associated with increased CRP in either obese individuals (CRP ratio 1.052, 95% CI 0.991 to 1.111, p = 0.1) or in non-obese older adults (CRP ratio 1.00, 95% CI 0.92 to 1.08, p = 0.99), mirroring the pattern of sarcopenia associations with pre-diabetes and diabetes.

## Discussion

As hypothesized, sarcopenic obesity was strongly associated with increased insulin resistance and dysglycemia. In addition, sarcopenia was associated with increased insulin resistance in both non-obese and obese individuals, and also associated with higher levels of HbA1C in obese individuals. Thus, sarcopenic obese individuals had significantly higher HOMA-IR and HbA1C levels than obese individuals without sarcopenia, confirming our hypothesis that the combination of sarcopenia and obesity leads to more severe insulin resistance and dysglycemia.

However, there were important differences in the effect of combined sarcopenia and obesity, by age. In those under 60 years of age, sarcopenia was strongly associated with more insulin resistance and higher HbA1C levels in both non-obese and obese individuals, and also associated with higher prevalence of diabetes in obese individuals. Thus, the younger sarcopenic obese individuals had significantly and markedly higher HOMA-IR and HbA1C levels and diabetes prevalence than younger obese individuals without sarcopenia. On the other hand, in those 60 years or older, although sarcopenia was associated with increased insulin resistance in both non-obese and obese individuals, sarcopenia did not add to the risk of insulin resistance or dysglycemia in obese older adults.

This marked age difference in the metabolic effect of sarcopenia is likely to be the result of differences in the etiology of sarcopenia in young compared with old individuals. While sarcopenia in young and middle-aged adults likely reflects reduced accumulation of skeletal muscle mass over the life course, in older individuals sarcopenia results from a combination of inadequate muscle mass accumulation when younger and reduction in muscle mass from peak levels when older. Skeletal muscle is a primary tissue responsible for insulin-mediated glucose disposal; thus in sarcopenia, the lower total mass of muscle should cause diminished insulin-mediated glucose disposal, independent of obesity. However, type II muscle fibers, which are less responsive to the metabolic actions of insulin [21], are lost to a greater extent than type I fibers in age-related muscle atrophy [10]. Thus sarcopenia due to age-related muscle atrophy could mean increased insulin sensitivity and more efficient glucose disposal [11]. This might explain the observed lack of association between sarcopenia and diabetes in non-obese older adults. However, decrease in type II muscle fibers has not been shown to improve overall myocellular insulin action in post-menopausal women [11] and to the contrary, recent reports show that increased type II fiber population improves glucose disposal in mice [7]. The role of fiber type distribution in age-induced insulin resistance remains controversial. In obese older adults however, there is greater lipid content within skeletal muscle [22], which is associated with diminished muscle insulin sensitivity [23]; this might in part, explain why sarcopenia did not confer protection from dysglycemia and diabetes in obese older adults (unlike in non-obese older adults). Further work is needed to illuminate the roles of fiber type distribution and intramyocellular lipid accumulation in age-related insulin resistance and diabetes.

Chronic low-grade inflammation is now recognized as a central mediator of obesity-associated insulin resistance [24]. Genetic and pharmacologic inhibition of inflammatory mediators is shown to prevent diet- and obesity-induced insulin resistance as well as prevent accelerated loss of muscle mass with age [25]. Our data also suggest that in young and middle-aged individuals (both obese and non-obese) sarcopenia is associated with greater inflammation (higher levels of serum CRP). This association was not seen in non-obese older adults. This pattern mirrors the strong association of sarcopenia with insulin resistance and dysglycemia in young adults, in contrast to the weaker association in older adults, suggesting that inflammation may have a role in the development of metabolic complications from sarcopenia.

Our study had some important limitations. The cross-sectional nature of the study limits our ability to draw causal inferences from the relationships observed. For instance, it is possible that diabetes and dysglycemia lead to sarcopenia and sarcopenic obesity. However, the strength of the observed associations and their persistence after exclusion of individuals with type 2 diabetes, bolster the case for sarcopenia and sarcopenic obesity causing insulin resistance and dysglycemia. Secondly, as NHANES III was conducted among the non-institutionalized U.S. population, and because participants who were physically unable to attend the mobile examination center were not included in our analysis, we may have underestimated the prevalence of sarcopenia. Finally, we used BI to estimate muscle mass, which may have led to some individuals being erroneously classified in or out of the sarcopenic obese category. However, such misclassification errors would only have weakened associations between sarcopenic obesity and insulin resistance or dysglycemia.

In conclusion, this large national study found that sarcopenic obesity, to a greater extent than sarcopenia or obesity alone, is strongly associated with insulin resistance in both young and old adults, underscoring the important role of low muscle mass as an independent risk factor for metabolic disease. In those under 60 years of age, sarcopenia also increased the risk of dysglycemia, in both non-obese and obese individuals. In young as well as in old adults, sarcopenia was also much more prevalent in obese than in non-obese individuals. With the ongoing obesity epidemic in the U.S. and the disturbing increases in the incidence of obesity in children and young adults, our data suggest that we can expect to see sharp increases in sarcopenia and diabetes in the coming years. In this environment, interventions aimed at increasing muscle mass in younger ages and preventing loss of muscle mass in older ages may have the potential to reduce type 2 diabetes risk. Further research is required to understand the pathophysiology and metabolic basis of the associations documented here, as well as to develop effective means of preventing sarcopenic obesity and its metabolic consequences.
